# ASO Author Reflections: Predictive Clinical Nomograms for Postoperative Outcomes Following Porto-Mesenteric Vein Resection with Pancreatectomy: Identifying the Best Patient for a High-Acuity Surgery

**DOI:** 10.1245/s10434-025-17833-5

**Published:** 2025-07-12

**Authors:** Shailesh V. Shrikhande, Deeksha Kapoor, Vikram A. Chaudhari, Manish S. Bhandare

**Affiliations:** Gastrointestinal and Hepato-Pancreato-Biliary Services, Department of Surgical Oncology, Homi Bhabha National Institute, Tata Memorial Hospital, Parel, Mumbai, Maharashtra India

## Past

The evolution of pancreatic surgery has been shaped by numerous formidable challenges, frequently accompanied by a prevailing sense of surgical nihilism. From the technical complexities of pancreatic anastomosis to the high-risk undertaking of vascular resections, each milestone was initially met with scepticism, underscoring the cautious yet persistent trajectory of progress in this demanding field. Although the first vein resection (VR) combined with pancreatectomy was performed as early as the 1950s, widespread acceptance of the procedure was delayed due to the historically poor prognosis associated with pancreatic adenocarcinoma (PDAC), the perceived high complication rate, and the limited benefit of systemic therapy.^1,2^ Over the years, it has become well established that when performed appropriately, aggressive surgery combined with systemic therapy plays a pivotal role in the treatment paradigm for pancreatic cancer. With growing experience in vascular resections, extended pancreatectomies and VRs have gained momentum and are now being performed with increasing frequency. The same trend has been observed in India, where the number of pancreatic tumors has traditionally been considered low.^3^ The key lies in offering a high-acuity operation to carefully selected patients who are likely to tolerate the procedure well and have the greatest potential for long-term survival. The complication rate of VR with pancreatectomy has decreased over the years, with a reported benchmark of 2.8% in-hospital mortality and 6.5% 5-years survival for PDAC.^4^ Although the overall complication rates in patients undergoing VR have been reported to be comparable with those not undergoing VR, segmental resections are consistently associated with a higher incidence of postoperative complications compared with tangential resections. Additionally, the success of systemic therapy demonstrated in the PRODIGE 2018 and ESPAC-4 trials has provided greater justification for undertaking aggressive surgical resections.

## Present

We presented multicentric data from India on VR combined with pancreatectomy and analyzed the factors influencing the development of 90-day postoperative mortality (POM) and major complications (MCs). These predictive variables were integrated into nomograms to explore their complex interplay and cumulative impact on postoperative outcomes. Survival outcomes in patients with PDAC were also examined.^5^ Over an 8-years period, 389 cases of VRs with pancreatectomy were reported from 11 experienced centers in pancreato-biliary surgical units in India for patients undergoing surgery for pancreato-biliary tumors, the study led by the Tata Memorial Centre, Mumbai. We reported postoperative outcomes consistent with the world literature, with a 30-day POM rate of 2.8%, a 90-days POM rate of 6.4%, and an MC rate of 32.6%. We found that a high Charlson’s comorbidity index, preoperative biliary drainage, preoperative radiation, segmental VR, and the need for additional organ resection were significant factors impacting 90-day POM. Similarly, a high American Society of Anesthesiology grade, preoperative radiation, and an additional organ resection impacted the MC rate. Segmental VRs were associated with higher failure-to-rescue rates rather than merely an increased incidence of MCs. These parameters were integrated into nomograms to predict postoperative outcomes following VR with pancreatectomy.

The overall survival (OS) and disease-free survival (DFS) outcomes reported for patients with PDAC were consistent with contemporary literature, with a median OS of 25.01 months and a median DFS of 16.72 months. Segmental VR, perineural invasion, and R1 resection margins were significantly associated with poorer OS and DFS. As anticipated, larger tumors negatively impacted OS. OS of radiologically borderline resectable and locally advanced PDAC was similar.

## Future

As the paradigm shifts from anatomical to biological resectability in PDAC, vascular involvement is increasingly regarded as a technical hurdle rather than a definitive contraindication to surgery.^6,7^ Moreover, existing definitions of anatomically borderline and localized PDAC demonstrate significant interobserver variability and offer limited prognostic distinction—a perspective that is now being actively questioned.^7,8^ These evolving insights are likely to encourage more aggressive surgical approaches in carefully selected patients, especially when disease biology is assessed and optimized through systemic therapy. We believe that comprehensive nomograms, such as those developed in this study, can play a pivotal role in identifying patients most likely to tolerate and derive meaningful benefit from these complex surgical procedures. By integrating diverse clinical and radiological variables, these tools enable personalized risk stratification, enhancing informed surgical decision making. The increasing availability of big data in clinical, radiological, and pathological spheres, alongside the expanding applicability of machine learning, will unveil powerful avenues to refine the prediction of postoperative outcomes and survival events. This will facilitate precision surgery and individualized treatment strategies that account for the multifactorial determinants of surgical and oncological success.
